# Pregnant migrant and refugee women’s perceptions of mental illness on the Thai-Myanmar border: a qualitative study

**DOI:** 10.1186/s12884-015-0517-0

**Published:** 2015-04-15

**Authors:** Gracia Fellmeth, Emma Plugge, Moo Kho Paw, Prakaykaew Charunwatthana, François Nosten, Rose McGready

**Affiliations:** Nuffield Department of Population Health, University of Oxford, Old Road Campus, Headington, Oxford UK; Centre for Tropical Medicine and Global Health, Nuffield Department of Medicine Research Building, University of Oxford, Old Road Campus, Headington, Oxford UK; Shoklo Malaria Research Unit, Mahidol-Oxford Tropical Medicine Research Unit, Faculty of Tropical Medicine, Mahidol University, Mae Sot, Thailand; Mahidol-Oxford Tropical Medicine Research Unit, Faculty of Tropical Medicine, Mahidol University, Bangkok, Thailand; Centre for Tropical Medicine, Nuffield Department of Medicine, University of Oxford, Oxford, UK

**Keywords:** Migration, Migrant, Refugee, Pregnancy, Mental health, Qualitative, Myanmar

## Abstract

**Background:**

Mental illness is a significant contributor to the global burden of disease, with prevalence highest in low- and middle-income countries. Rates are high in women of childbearing age, especially during pregnancy and the first year post-partum. Migrant and refugee populations are at risk of developing mental illness due to the multiple stressors associated with migration. The Thai-Myanmar border area is home to large populations of migrants and refugees as a result of long-standing conflict, poverty and unemployment in Myanmar. This study aims to explore perceptions of mental illness among pregnant migrants and refugees and antenatal clinic staff living and working along the Thai-Myanmar border.

**Methods:**

Thirteen focus group discussions were conducted with pregnant migrants, pregnant refugees and antenatal clinic staff. Focus groups were held in one large refugee camp and two migrant health clinics along the Thai-Myanmar border. Thematic analysis was used to identify and code themes emerging from the data.

**Results:**

A total of 92 pregnant women and 24 antenatal clinic staff participated. Discussions centered around five main themes: symptoms of mental illness; causes of mental illness; suicide; mental illness during pregnancy and the post-partum period; and managing mental illness. Symptoms of mental illness included emotional disturbances, somatic symptoms and socially inappropriate behavior. The main causes were described as current economic and family-related difficulties. Suicide was frequently attributed to shame. Mental illness was thought to be more common during and following pregnancy due to a lack of family support and worries about the future. Talking to family and friends, medication and hospitalization were suggested as means of helping those suffering from mental illness.

**Conclusions:**

Mental illness was recognized as a concept by the majority of participants and there was a general willingness to discuss various aspects of it. More formal and systematic training including the development of assessment tools in the local languages would enable better ascertainment and treatment of mental illness in this population.

## Background

Mental illness is a significant contributor to the global burden of disease. Worldwide, mental illness and substance use represent the leading cause of years lived with disability and the fourth leading cause of overall disease burden as measured by disability adjusted life-years [1]. This burden is set to increase further due to the substantial co-morbidity of mental illness with other chronic conditions such as cancer, cardiovascular disease and diabetes which are becoming ever more prevalent [1,2]. Yet mental health remains a neglected field. Globally, the burden of mental illness falls most heavily on low- and middle-income countries [3]. In these settings, resources to appropriately diagnose and manage mental illness are particularly scarce as communicable diseases and emerging non-communicable diseases such as cancer and cardiovascular disease are prioritised as more ‘urgent’ [1]. Across low-income countries the treatment gap is estimated to be as high as 90% due to a lack of mental health resources and facilities, inequities in their distribution and inefficiencies in their use [1,4,5].

Women are at greater risk than men of experiencing mental disorders, and amongst women rates are highest during childbearing years [1,6]. The perinatal period in particular is a time at which women are at increased risk of developing mental illness, with rates up to three times higher than in other periods of women’s lives [6,7]. The mechanisms for these increased rates are likely to be a combination of social, psychological and biological factors [8]. Prevalence of perinatal mental disorders range between 10-15% in high-income countries compared with to 10-41% in low and middle-income countries [6,9,10]. Mental illness during pregnancy and post-partum significantly affects not only the mother but also the child’s physical and psychosocial development, family relations and wider society [3]. Addressing the mental health status of these women is directly relevant to the United Nations’ Millennium Development Goals of reducing child mortality (MDG4), improving maternal health (MDG5) and promoting gender equality and empowering women (MDG3).

Other groups who are known to be at increased risk of poor mental health are those living in poverty and socially marginalised groups [11,12]. This includes migrants and refugees who are at high risk of developing mental illness as a result of past and on-going hardships [13,14]. Migration is known to be a highly stressful process of adjustment with significant effects on emotional health [15]. Evidence suggests that migrants and refugees experience higher rates of mental illness than host populations [14,16,17].

 Of the 214 million migrants globally, the vast majority relocate within low and middle-income regions where local resources may already be stretched [18,19]. Within Southeast Asia, Thailand is a major recipient of individuals fleeing long-standing conflict and extreme poverty in neighbouring countries. In Myanmar, civil war between the government and a number of independent ethnic groups has been ongoing since the 1960s, leading to the displacement of large populations of ethnic minority groups to Thailand. There are an estimated 200,000 migrants and 120,000 refugees currently living and/or working along the Thai-Myanmar border [20-22].

Myanmar’s refugees and migrants in Thailand constitute two major groups. Refugees include persons fleeing violence and conflict arising from ethnic, political and religious persecution [18,20]. Those who enter Thailand live within established refugee camps on the Thai side of the border. The Myanmar refugee context in Thailand is one of the world’s major protracted refugee situations [21,23]. Traumatic past exposures act as risk factors for mental disorders such as depression, anxiety and post-traumatic stress disorder. For many, including those born in the camps, the intractable “state of limbo” and unfulfilled economic, social and psychological needs pose an additional risk factor for poor psychological health [23,24].

Migrants, on the other hand, have often left Myanmar primarily as a result of longstanding conditions of poverty and unemployment. Attracted by Thailand’s relative political stability, greater employment opportunities and wages up to ten times higher than in Myanmar, migrants tend to live in villages or temporary shelters on either side of the border, often making daily commutes into Thailand for work [20]. Conditions upon arrival vary and details of variation for urban versus rural migrants have not been explored. At one extreme, migrants may end up in ‘dirty, dangerous and demeaning’ jobs unwanted by the local Thai population and experience exploitative working conditions, unsanitary living conditions, discrimination and limited access to government healthcare and other social services [20,25,26]. The constant threat of deportation for migrants without documentation can create an additional source of chronic stress [27]. However, other migrant workers find stable employment with reasonable working conditions which enable them to send regular remittances to relatives in Myanmar or to live as family units in Thailand or close to the border. Some employers provide registration and health insurance coverage for migrant workers. For some migrants, therefore, even though daily hardships remain, life may be better than it was in Myanmar.

Migrants and refugees typically live in bamboo housing with water collected from communal wells. Pit toilets are usually shared by a couple of households. Health services on the Myanmar side of the border are scarce and access to Thai government services low due to lack of entitlements as well as language and transportation barriers [28]. Medical and antenatal services are provided by non-governmental organisations. Mental health services available to Myanmar migrants in Thailand are limited to emergency care and not optimal given the vast difference in languages. In Mae Sot, two non-governmental organisations and a healthcare clinic for migrants offer mental health services. As these services are all relatively new it is not possible to determine at this stage if there is stigma around help-seeking or a lack of awareness of services, and similarly whether mental health is under-reported or under-diagnosed.

This study was conducted to explore the feasibility and acceptability of carrying out a more extensive, questionnaire-based study to assess prevalence of perinatal depression. The aim of this current study was to establish pregnant migrant and refugee women’s perceptions and understanding of mental illness, their experiences and beliefs with regards to mental illness, and their willingness to discuss mental illness in a focus group setting. We also included antenatal clinic (ANC) staff as we felt it was important to ascertain whether staff who would potentially be involved in administering questionnaires as part of a prevalence study were familiar with the concept of mental illness and felt comfortable discussing it. We were interested in mental illness in general, though some questions related specifically to depression as one of the commonest mental disorders. Results will be used to inform the design and methods of the planned prevalence study.

## Methods

### Setting

**Figure 1:**
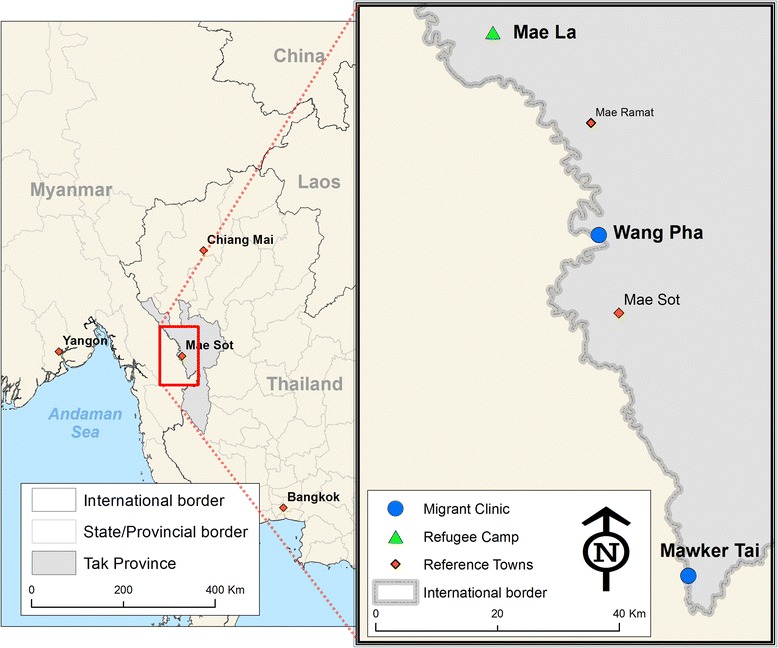
1 Map of study area showing refugee (▲) and migrant clinics (●) (Credit to Daniel M Parker, SMRU).

This study was conducted at three outreach ANCs run by the Shoklo Malaria Research Unit (SMRU) to the north and south of Mae Sot, Thailand. SMRU has provided free medical and obstetric services within the Thai-Myanmar border area since 1986 and has extensive experience of working with the local refugee and migrant populations [29]. SMRU ANCs are well attended and in 2010 more than 75% of deliveries occurred within SMRU clinics [28]. The three sites were a refugee clinic within Maela refugee camp (MLA) and two migrant clinics at Mawker Tai (MKT) and Wang Pha (WPA) (see Figure 1) [30].

### Participants

The population served by SMRU consists of migrants and refugees from Myanmar living along the Thai-Myanmar border. The local population is mostly of Karen ethnic background, has Christian, Buddhist, Muslim and animist religious beliefs and speaks up to five different languages and dialects including Sgaw Karen, Po Karen, Burmese, Thai and English [31,32]. Maela is the largest established refugee camp along the border with a population of 43,000 [33]. Migrants live in villages along both sides of the border. The literacy rate is 50% amongst pregnant women [29]. This study focused on pregnant refugee and migrant women attending ANC at MLA, MKT and WPA and the locally-trained staff providing their antenatal care.

### Data collection

Qualitative methods were used to elicit participants’ perceptions of mental illness. Focus group discussions (FGD) used convenience samples of pregnant women and SMRU antenatal clinic staff.

#### Pregnant women

All pregnant women attending refugee (MLA) and migrant (MKT, WPA) antenatal clinics during the period of the research were invited to participate. An announcement about the research was made by an ANC staff member to women in the waiting area. Those interested in taking part were given further information about the purpose of the research and the topic of discussion in a separate room adjacent to the waiting area. Participation was voluntary and women were informed that if they chose to take part they could leave the discussions at any time without needing to provide a reason. Of those women who attended the adjacent room to obtain further information, all went on to take part in the discussions. Ten focus groups of between 4-11 women were conducted across the three sites. Women were separated into groups on the basis of language spoken (Burmese or Karen).

#### Antenatal clinic staff

All antenatal clinic staff who were rostered onto daytime duty during the period of the research were invited to participate. A total of three focus groups (one at each site) with ANC staff were conducted, each consisting of between 4-12 women depending on staff availability. Recruitment methods were the same as for pregnant women. Although some staff spoke English, discussions were conducted in Karen in order to ensure all could understand and participate in discussions equally well.

#### Setting

FGDs were conducted in private areas adjacent to ANC waiting areas with participants sitting in a circle on the floor or around desks as is typical in these settings [34]. Each FGD lasted between 20-40 minutes. Discussions were led by one author (MKP) who has lived in a refugee camp for more than 25 years, completed her training in the camp [35] and worked with pregnant women from the local population but was not personally known to the FGD participants. This midwife is fluent in Karen, Burmese and English and has extensive experience in conducting FGDs. Another author (GF), who was not known to participants and not involved in their clinical care, was also present. The midwife leading the FGDs translated participants’ responses into English during the course of the discussions whilst GF made written notes. Women are used to on-going translation in the clinic setting as required. In order to maintain confidentiality, no names were recorded on paper. Each participant received a bar of soap at the end of the discussion as a token of thanks. FGDs were conducted on three consecutive days in January 2014.

FGDs were guided by questions aimed to elicit participants’ perceptions around mental illness. Questions were translated into Karen and Burmese by the midwife who led the FGDs. Translated versions were back-translated by another staff member at SMRU to ensure that the meanings of individual questions had been maintained.

Questions were phrased in a way that avoided participants being asked directly to share personal experiences. This approach was adopted so that participants would not feel pressured into divulging information that they may not want to within a group setting. By using more general, open-ended questions, women were free to discuss personal experiences only if they wanted to. The questions listed below were used as a prompt to guide discussions.

1. Have you heard of illnesses of the mind?
2. Have you heard of an illness where people feel very sad for a long time?
3. Have you heard of an illness where people are so sad they want to die?
4. Do you think these illnesses happen often? In 100 people, how many do you think will be feeling very sad?
5. Why do you think these illnesses happen?
6. Have you heard of these illnesses happening in pregnant/post-partum women? Do you think it happens more or less often in these women and why?

### Data analysis

Data from FGDs were reviewed at the end of each day of data collection. Systematic data analysis occurred after data collection was complete. Data saturation was not formally assessed during the data collection period. Data from the FGDs were analysed using *NVivo 9*. Thematic analysis was used to identify and code themes emerging from the data [36,37]. Two authors (GF and EP) independently analysed and coded data before comparing and discussing themes identified.

### Ethics and consent

Prior to starting each FGD a verbal explanation of the study was provided. Those who agreed to take part were asked to sign or thumbprint a consent form which was read out in Karen or Burmese by the facilitator. Ethical approval for the study was obtained from the University of Oxford’s Tropical Research Ethics Committee (OXTREC 1056-13) in December 2013 and from the Tak Border Community Advisory Board (T-CAB) in January 2014. The T-CAB consists of community representatives who review the ethics as well as the applicability, relevance and appropriateness of research proposals involving the local population.

## Results

A total of 92 pregnant women (29 refugees and 63 migrants) participated in ten focus groups, of which four were conducted in Karen and six in Burmese. Twenty-four ANC staff participated in three focus groups, all of which were conducted in Karen. No participants left during any of the FGDs. Themes emerging from the FGDs fell into five key areas: symptoms and behaviours related to mental illness; causes of mental illness; explanations and reasons for suicide; mental illness during pregnancy and the post-partum period; and managing mental illness.

Overall, responses did not differ between refugee (MLA) and migrant (MKT and WPA) women. There were some differences between ANC staff and pregnant women in the levels of awareness around mental illness: approximately two-thirds of participating pregnant women had heard of ‘illnesses of the mind’ compared with all of the ANC staff, for example. Differences were also evidenced by the depth of discussions, the number of examples and anecdotes provided for topics being discussed and general levels of engagement in discussions. However, the overall themes that arose were common across all focus groups and there were very few differences between pregnant women and ANC staff in terms of discussion content. As this study is focused on perceptions relating to mental illness rather than depth of knowledge, results of staff and patient FGDs were analysed together. Any differences between groups are highlighted.

1. *Symptoms and behaviours related to mental illness*

### Loss of control over emotions

The most commonly described symptoms associated with mental illness were emotional and mood disturbances including feelings of intense sadness, anger and fear. Emotions were frequently characterized as taking over the individual and described alongside an element of lack of control. *“Crying for no reason”* and *“wanting to shout for no reason”*, for example, were cited by respondents as key characteristics of individuals with mental illness. One participant described an individual she knew who had a mental illness as:

*“Getting angry very easily for no reason and acting aggressive(ly) or wanting to hit other people”* (Pregnant refugee; FGD 3)

Another participant associated mental illness with a labile emotional state, again emphasizing a sense of loss of control. She described having:

*“Different emotions all the time: sometimes feeling angry, then sad, then quiet”* (Pregnant migrant; FGD 7)

### Inappropriate social behaviour

Many participants associated mental illness with social behaviour considered unusual or inappropriate within the local cultural and social context. For example, individuals with mental illness were described as wanting to spend time alone and without talking to other people (*“closing the door and staying by oneself”*; pregnant migrant; FGD 7) – a trait considered atypical in a society with a strong sense of community and social interaction. The idea of mental illness manifesting itself as socially deviant behaviour arose in examples of individuals going out at inappropriate times, for example late at night, and without purpose (*“going out wandering everywhere without reason”*; pregnant migrant; FGD 8). Often, individuals with mental illness were described as acting “crazy” or in a manner that made their illness very visible to others:

*“One man I knew was always making strange gestures with his hands like he was counting something on his fingers and smiling to himself for no reason”* (Pregnant migrant; FGD 10)

### Excessive worry

Excessive worry or “thinking too much” was mentioned frequently as a behavior related to mental illness. Out of the ten FGDs with pregnant women, four groups mentioned *“thinking too much”* or *“thinking a lot”* as symptoms of mental illness. Interestingly, it was also described as a cause of mental illness.

### Somatic symptoms

Several physical symptoms were associated with mental illness, including headaches, loss of appetite, poor or excessive sleep, heart palpitations and having cold hands and feet. One participant described a feeling of a *“very big and heavy”* head. Another described a man she knew as having:

*“Muscle spasms, with his fingers and toes curling up and a feeling of a very tight and tense body. He had to have a massage to relax the muscles.”* (Pregnant migrant; FGD 4)

2. *Causes of mental illness*

### Economic, family and domestic issues

Lack of employment and low income were commonly cited causes of mental illness, and these overlapped with domestic and family-related issues. Unsupportive partners, parents and other family members were regarded as causing strained relationships which were exacerbated by low household incomes.

### Excessive worry

As well as being a commonly cited symptom, excessive worry was also frequently described as a cause of mental illness. When asked what might cause mental illness, typical responses included:

*“Thinking too much about the future and possible problems”* (Pregnant migrant, FGD 5)

*“Worrying about family and children”* (Pregnant migrant, FGD 6)

### Spirits

The idea that spirits can cause mental illness was raised only by a very small number of participants, including one member of ANC staff. It did not appear to be a commonly held belief, and the times at which participants mentioned it did not spur others on to either agree or disagree. One participant felt that:

*“Maybe bad spirits cause (mental illness) – for example, if someone is jealous of a woman and puts a spell on her, then that women could become crazy”* (Pregnant migrant; FGD 9)

Spiritual and animistic beliefs were sometimes described as something that others, but not respondents themselves, believed:

*“Some Karen believe that if the mother touches the first rain then she will develop mental illness”* (Pregnant refugee; FGD 2)

### Trauma

One single participant raised the idea that a specific event or trauma could lead to mental illness:

“*One girl I knew was working in a factory and witnessed a fire. After that she became very nervous all the time and was doing dangerous things without thinking, for example crossing a busy road with many cars without looking.”* (Pregnant migrant; FGD 6)

3. *Suicide*

Shame emerged as a significant theme in discussions around suicide. Other common elements were economic hardships, domestic and family issues. These were often described as factors which brought shame upon an individual and led to suicide.

“*One girl I knew killed herself because she lost some expensive jewelry and felt ashamed when her family was angry with her”* (Pregnant refugee; FGD 1)

*“A schoolboy in the village aged 13 or 14 years took weed-killer because he couldn’t pay back some money he owed”* (Pregnant migrant; FGD 4)

*“One boy from the village aged about 13 years, his parents were angry with him and hit him with a stick. He felt very sad and ashamed and killed himself by hanging.”* (Pregnant migrant; FGD 6)

*“In one case, the husband was an alcoholic and the wife felt ashamed so she committed suicide”* (ANC staff; FGD 11)

Interestingly, suicide was not seen so much as an endpoint or extreme manifestation of mental illness but more as a separate condition. The causes that were mentioned – including shame, guilt, economic and family issues and spiritual causes – suggest that suicide was not necessarily attributed to mental illness.

4. *Mental illness in pregnancy and the post-partum period*

### Lack of family support

Participants described mental illness as occurring more frequently during pregnancy and the post-partum period. A common theme was the lack of emotional, practical and financial support offered by family and partners. Many women described partners’ lack of involvement in childcare meaning that the new mother was often left feeling alone and overwhelmed.

“*The husbands don’t take responsibility for the household and food so women can’t eat and are not taken care of”* (ANC staff; FGD 11)

### Worry

Another reason cited for higher levels of mental illness in the post-partum period was women worrying about their children. Participants described new mothers not knowing how to take care of the children, especially given the lack of support from family, but also worrying about the longer-term future and opportunities for their children.

*“You have to think about your children’s future and you have more to worry about”* (Pregnant refugee; FGD 3)

5. *Managing mental illness*

### Social and emotional support

Social and emotional support in the form of talking and encouragement from friends, family and neighbours was the most commonly suggested means of managing mental illness. Several participants mentioned that as a listener, one should offer empathy but also encouragement. A number of participants also discussed the value of providing practical support such as helping affected individuals to re-engage in social life and find employment:

*“If they don’t have a job then we can help them find a job”* (ANC staff; FGD 12)

*“Giving them some work to do because if they don’t have work they think too much”* (ANC staff; FGD 13)

*“Helping them to participate in activities”* (ANC staff; FGD 11)

### Counselling

While pregnant women and ANC staff were equally likely to suggest talking informally to friends and family, only ANC staff mentioned talking to healthcare workers and, more specifically, counseling (including referral to an existing counseling service) as a means of helping those with mental illness.

### Medication and hospitalization

Taking medication and being admitted to hospital were invariably seen by both pregnant women and ANC staff as other helpful options for managing mental illness. Often they were regarded as a more extreme form of treatment, reserved for cases in which social support and talking therapies had not been effective:

*“If talking is not enough then sometimes you need medication”* (ANC staff; FGD 12)

### Prayer

The use of prayer to help alleviate the mental illness of others was mentioned only once by one ANC staff member.

## Discussion

This study of pregnant migrant and refugee women and ANC staff on the Thai-Myanmar border used qualitative methods to elicit participants’ perceptions around mental illness. Although ANC staff had more in-depth awareness and experiences of dealing with mental illness, discussions of both ANC staff and patients revealed similar content and themes. This common cultural understanding of mental illness among staff and patients is important as it suggests there is a positive way forward for treating and managing those affected. There were also no differences elicited between refugee and migrant women, nor between groups conducted in Karen or Burmese. Participants identified a number of emotional, behavioural and physical manifestations of mental illness, and agreed that economic, family and domestic issues often contributed to these illnesses. They believed that mental illness occurred more commonly during pregnancy and the post-partum period, attributing this to the lack of emotional, practical and financial support offered by family and partners. Many participants believed that mental illness is best managed by emotional and social support from friends and family although some identified input from health care staff and medication as helpful.

The fact that responses did not differ significantly between refugee and migrant women suggests that similarities (such as both groups’ predominantly Karen background) are perhaps greater than differences (such as migrant versus refugee status). It is possible that differences were not elicited due to the indirect phrasing of questions (“have you heard of…”). If participants had been asked more directly about personal histories of mental illness, variations in the experiences of migrants and refugees may have surfaced. Further studies need to explore the background of women and their families in more detail to understand how they have come to attend or work in ANCs in refugee and migrant communities. The relationship between mental health status and length of residence in Thailand would also be an important factor to explore further.

Participants were willing to talk about mental illness and generally engaged positively in discussion. This in itself is an important observation in a society where mental illness remains ‘taboo’. It suggests that if conducted in a sensitive manner, ideas and experiences around mental illness can be elicited and used constructively to inform solutions. In a number of groups, and especially amongst the ANC staff, discussions seemed to provide a welcome (and presumably rare) opportunity for participants to share experiences and thoughts around mental illness. A similar observation was made in a study of the general health of Karen refugees resettled in the USA, which found that contrary to expectations, the topic of mental illness generated more discussion than any other aspect of health [38].

The symptoms most commonly associated with depression by participants were mood disturbances such as feelings of sadness, anger and fear. These are similar to classic symptoms described in the ‘biomedical’ model of psychiatric illness. However, emphasis was also placed on the social implications of these mood states. Social isolation and wanting to be alone were regarded as important sequelae of these abnormal emotional states, as was social disinhibition in the form of going out late at night and without reason. Seeking privacy may also be considered a reaction to the lack of privacy in highly crowded refugee camps and some of the temporary migrant shelters. Another study of Karen medics working in conflict zones within Eastern Myanmar reports similar findings: participants associated depression with social isolation and feelings of futility. The somatic symptoms described by our participants also reflect findings of other studies of Karen and Karenni communities in Thailand or within Myanmar, which have included loss of appetite, excessive sleep, trembling, palpitations, numbness, the heart “feeling tired”, “feeling hot under the skin”, and “thinking too much” [39,40].

Amongst the causes of mental illness, current economic and family-related causes were the most commonly raised issues. The absence of discussion about past traumas was striking in a population exposed to high levels of stressors prior to migration. In Power’s study of Burmese refugees in the USA, for example, depression was attributed to the lingering impact of having lived in refugee camps, past traumas and physical and psychological hardships endured, as well as ongoing social and cultural isolation following resettlement [38].

There are many possible explanations for past traumas not being mentioned by our participants. First, FGDs and/or the questions posed may not have been conducive to eliciting such information. Participants may have discussed these issues more had they been given more time, opportunity or encouragement to do so. They may have felt uncomfortable discussing their past in the presence of friends, colleagues or the researchers. Another possibility is that our findings reflect the age group of participants, who may have left Myanmar under less traumatic conditions or at too young an age to remember any trauma. However, this is unlikely to explain our findings fully. A third possibility is that past traumas are genuinely not considered relevant to current mental health status by this group. The local population may represent a particularly resilient group, or past experiences may deliberately be suppressed to preserve energy for continuing hardships. More in-depth research is required to explore whether cultural attributes such as collectivism or community cohesiveness within this particular ethnic group of Myanmar women might act as a protective factor [41]. Finally, on-going stresses – such as the economic and family-related difficulties raised by the participants and worries about future – perhaps dominate the concerns of this group. A more thorough understanding of participants’ backgrounds is required to better understand this important negative finding.

With regards to the management of mental illness, social support from friends and family was commonly cited as were more formal means such as medication and hospitalisation. ANC staff were more likely than pregnant women to mention the benefits of talking to medically trained staff or counsellors, though such trained counselling staff are in extreme shortage. These findings are similar to those described in other studies assessing coping strategies among Myanmar refugee populations [38,40]. The fact that only one mention of prayer and no mention of animistic mechanisms of healing were made is also interesting. Again, further research is required to explore whether this is because these factors are not considered important or whether participants were reluctant to talk about them.

### Strengths and limitations

This study benefits from a number of strengths. First, to our knowledge it is the first investigation of the perceptions of mental illness from the perspective of pregnant and refugee migrant women in the local area. As rates of mental illness are at their highest among pregnant and post-partum women, the views of this group are particularly important.

A further strength of this study is its use of FGDs. Qualitative methods including FGDs are especially useful in the exploration of new and relatively under-researched fields of study [36]. They provide effective means of eliciting and sharing information especially on sensitive topics, and evidence suggests they work particularly well with women [42]. Kitzinger found in her work on Acquired Immunodeficiency Syndrome (AIDS) in the 1980s and 1990s that despite sensitive topics such as sexual behavior being discussed, less inhibited members of the group ‘broke the ice’ for the more reticent and this seemed to be the case particularly with groups who might express feelings common to the group but outside mainstream culture (or the assumed culture of the interviewer) [42,43]. The presence of two researchers for up to ten participants shifts the balance in favour of participants and empowers them [43]. FGDs have been used in the Thai-Myanmar border population for many years [44] and are a culturally-accepted method particularly suited to low-literacy populations [31,32]. The running of these FGDs in themselves will have created a certain level of awareness amongst participants and others of mental illness in the population.

There are also a number of limitations of this study. First, the presence of a foreign and English-speaking researcher may have influenced participants’ responses [36,38]. The use of an interpreter to facilitate discussions runs the risk of misrepresentation or bias of responses [38,40]. However, given that the interpreter in this study was a midwife with extensive clinical and research experience who was also involved in the development of the guiding questions, potential negative effects were minimized. Ideally discussions should have been audio-recorded and transcribed [36] but due to limited resources and time constraints results were recorded in note form only.

Second, groups were not stratified by age, gravidity, ethnicity or religion. As older people and those in greater authority positions are highly respected in this setting, younger, first-time mothers may have felt intimidated and spoken out less in the presence of older, more experienced mothers [34]. The views of multigravidae women therefore may be over-represented.

Third, no demographic information on participants was collected and therefore we cannot say how representative our sample was in terms of age, parity, educational level and migration history, all of which may impact perceptions of mental illness. Ascertaining this individual-level information, however, may have aroused anxiety among participants who represent a vulnerable group, many of whom have ‘illegal’ status in Thailand. Women attending ANC and therefore able to participate in our discussions may also have greater levels of health literacy than women who do not access antenatal services, and therefore findings may not be generalizable to all migrant women in this area. However, given that health literacy in women attending is generally low at around 50% [29], we suspect the results to remain robust. As this research was designed as an exploratory study we were not so much concerned about having a representative sample but rather wanted to establish general perceptions around mental illness from those willing to engage in discussions. Individual interviews may provide a more appropriate context for eliciting more personal experiences of what remains a stigmatized condition.

Fourth, the number of FGDs conducted and the number of participants was determined by pragmatic considerations (including the amount of staff time available in a busy clinic setting and the number of pregnant women attending ANC on the dates of data collection) rather than achieving data saturation. However as this was exploratory qualitative work, ensuring key themes were identified was our main priority.

Finally, cultural factors related to the data collection may have influenced results. For instance, culturally-bound interpretations or manifestations of mental illness may have been missed if we did not use the right language or terminology to describe conditions we wanted to discuss [40]. Furthermore, the presence of a non-local researcher (GF) may have affected discussions. For example, a perception amongst participants that researchers might be dismissive of local spiritual beliefs may have negatively influenced their willingness to discuss this as an explanatory mechanism for mental illness.

## Conclusion

Mental health services on the Thai-Myanmar border are lacking, due in part to the health agenda being dominated by what are considered more acute or ‘urgent’ conditions including infectious diseases such as malaria and HIV. This is unsurprising given that malaria was solely responsible for a maternal mortality of 1,000 per 100,000 in 1986 [28] and the explosive HIV epidemic in Thailand and Myanmar. As these diseases have been brought under control, non-infectious chronic conditions such as cancer, cardiovascular disease and diabetes have moved higher up the agenda. We argue that given the epidemiological shift and the high level of risk factors the local population has experienced, mental health deserves greater attention and resources. Although the findings suggest a baseline level of awareness with regards to mental illness, especially amongst ANC staff, more formal and systematic training including the development of diagnostic tools in Karen and Burmese language, would enable more cases of mental illness to be picked up and treated earlier.

This is one of the few mental health studies conducted in Myanmar refugees and migrants living in Thailand and, to our knowledge, the first to focus on pregnant and post-partum women. Our findings provide an initial insight upon which to base further investigation of this under-researched aspect of health in a vulnerable population group. Mental illness poses an important challenge for health systems globally, and an improvement in population health is only possible if prevention and treatment of mental illness is included as a priority public health issue [1]. Establishing the prevalence of depression in pregnant and post-partum migrants and refugees is essential for the development of appropriate mental health services. Addressing the wider social and economic determinants of health, for example by improving living and working conditions, are especially important in this context. More specifically, effective and inexpensive prevention and treatment programmes are available that have been proven to work in resource-limited settings [45]. Their existence, alongside the ever-increasing global migration flows, render a better understanding and more systematic addressing of the mental health needs of displaced populations an urgent priority.

## Abbreviations

ANC: Antenatal clinic
FGD: Focus group discussion
MLA: Maela refugee camp
MKT: Mawker Tai migrant clinic
WPA: Wang Pha migrant clinic
